# A simple plug-in bagging ensemble based on threshold-moving for classifying binary and multiclass imbalanced data

**DOI:** 10.1016/j.neucom.2017.08.035

**Published:** 2018-01-31

**Authors:** Guillem Collell, Drazen Prelec, Kaustubh R. Patil

**Affiliations:** a MIT Sloan Neuroeconomics Lab, Massachusetts Institute of Technology, Cambridge, MA 02139, USA; b Department of Economics, Massachusetts Institute of Technology, Cambridge, MA 02139, USA; c Brain & Cognitive Sciences, Massachusetts Institute of Technology, Cambridge, MA 02139, USA; d Computer Science Department, KU Leuven, Heverlee 3001, Belgium; e Institute of Neuroscience and Medicine, Brain & Behaviour (INM-7), Research Centre Jülich, Jülich 52425, Germany

**Keywords:** Imbalanced data, Binary classification, Multiclass classification, Bagging ensembles, Resampling, Posterior calibration

## Abstract

Class imbalance presents a major hurdle in the application of classification methods. A commonly taken approach is to learn ensembles of classifiers using rebalanced data. Examples include bootstrap averaging (bagging) combined with either undersampling or oversampling of the minority class examples. However, rebalancing methods entail asymmetric changes to the examples of different classes, which in turn can introduce their own biases. Furthermore, these methods often require specifying the performance measure of interest *a priori,* i.e., before learning. An alternative is to employ the threshold moving technique, which applies a threshold to the continuous output of a model, offering the possibility to adapt to a performance measure *a posteriori*, i.e., a *plug-in* method. Surprisingly, little attention has been paid to this combination of a bagging ensemble and threshold-moving. In this paper, we study this combination and demonstrate its competitiveness. Contrary to the other resampling methods, we preserve the *natural* class distribution of the data resulting in well-calibrated posterior probabilities. Additionally, we extend the proposed method to handle multiclass data. We validated our method on binary and multiclass benchmark data sets by using both, decision trees and neural networks as base classifiers. We perform analyses that provide insights into the proposed method.

## 1 Introduction

Dealing with a class imbalance in classification is an important problem that poses major challenges [1]. Imbalanced data sets frequently appear in real-world problems, such as in fault and anomaly detection [2], [3], fraudulent phone call detection [4] and medical decision-making [5], to name a few. Standard learning algorithms are often guided by global error rates and hence may ignore instances of the minority class, leading to models biased towards predicting the majority class. Several methods have been proposed to alleviate this problem (see, e.g., [6], [7] for reviews). Often, a first choice consists of preprocessing the data by resampling to balance the class distribution [8], [9]. This is often achieved by either randomly oversampling (ROS) the minority class [9] or randomly undersampling (RUS) the majority class [10]. More sophisticated methods that generate synthetic minority class instances are also a popular choice, e.g., the synthetic minority oversampling technique (SMOTE [9]). We will collectively call these data preprocessing methods as rebalancing mechanisms as they, in general, aim to make the training data more balanced. This will also avoid confusion with other resampling mechanisms, e.g., the simple bootstrap. Rebalancing is often combined with ensembles as they show superior performance to a single classifier [11]. Many such combinations have been shown to be effective for imbalanced data classification [6], [12], [13]. However, there are several potential drawbacks of rebalancing methods: (1) potential loss of informative data when undersampling, (2) changes in the properties of the data, such as asymmetric changes in the density of examples of different classes, which in turn can cause the models to induce unwanted biases, e.g., miscalibrated posterior probability estimates [14], [15], (3) it is often not evident which class distributions to use for a given dataset and a performance measure of interest [16] (wrapper methods [17] can be employed to tune the model for a given measure, but they are computationally expensive and often cater towards only a single measure, e.g., either accuracy or F1-score), and (4) it is nontrivial to extend the sampling heuristics normally defined for binary data to multiclass data as there can be multiple minority/majority classes [18].

Moving decision thresholds is another technique to deal with class imbalance. The main difference between rebalancing and threshold-based methods is that the former relies on data preprocessing before learning happens, whereas the latter relies on manipulating the continuous output of a learned model, e.g., class weights or posterior probabilities. Among other proponents, Provost [19] advocated for threshold-moving as a method to deal with class imbalance. Nevertheless, surprisingly, little attention has been paid to this technique, often to an extent that it is not even considered for comparison when new methods are proposed.

While this technique has been utilized in combination with some popular learning methods including a small ensemble [19], [20], [21]. However, to our knowledge, the combination of threshold-moving with a bagging ensemble has not been thoroughly investigated. As is evident, threshold-moving depends on reliable continuous estimation of the output; therefore, bagging ensembles are a good candidate to combine with threshold-moving as they are known to provide good probability estimates [22], [23]. In this work, we study threshold-moving combined with bagging ensembles and show that it is a competitive method with several advantages.

In particular, we seek a method that provides well-calibrated posterior probability estimates. An important advantage of such a method is that it can be utilized as a plug-in method where the threshold can be set *a posteriori*, i.e., at the test phase. This provides an opportunity to achieve good performance on different measures using the same model [24]. This is a major improvement over other methods, e.g., cost-sensitive methods and rebalancing, which require the performance measure of interest to be specified at the learning phase. Here, we propose *Probability Threshold bagging* (PT-bagging) that, as we will show, passes as a *plug-in* method. The main motivation behind PT-bagging is to leverage the advantages of bagging while avoiding the problems that rebalancing methods inevitably entail, as described above. The proposed method PT-bagging addresses those problems and possesses several desirable properties:

(1) It is a plug-in method that maximizes a performance measure of interest without retraining, but rather by just applying an appropriate threshold *a posteriori*. By contrast, rebalancing methods are not flexible and need computationally expensive parameter tuning, e.g., to find which class proportions to use for learning via a wrapper approach [17].
(2) It consistently performs close to the best possible macro-accuracy and macro F1 performances without the need to empirically find the optimal threshold (e.g., by cross-validation). Obtaining a validation set for tuning can be computationally costly, might not always be possible, or might be financially prohibitive (e.g., due to data collection costs).
(3) It can be extended to handle the multiclass setting when appropriate thresholds for a performance measure of interest are available, e.g., macro-accuracy.

We provide a theoretical analysis on when optimal macro-accuracy performance is guaranteed. However, for other measures, such as the macro F1-score, it is not always possible to obtain a closed-form expression for the optimal thresholds [25]. Nevertheless, we show that our new, simple and sensible threshold is close to the optimal threshold, and that PT-bagging achieves higher macro F1-score performance compared to other methods. In this respect, we make two additional contributions: (1) the proposal of a threshold for maximizing the macro F1-score, and (2) a comparison and analysis of the *full potential* of the methods, which we define as their maximum attainable performance if the optimal threshold were known.

The rest of this paper is organized as follows: in Section 2 we provide the relevant background, describe some popular resampling methods, and discuss their potential flaws. In Section 3, we describe our proposed method, PT-bagging, and provide a theoretical justification of its performance. In Section 4, we describe our experimental setup. In Section 5, we present a comprehensive set of empirical tests and discuss the results. Finally, we comment on the implications of our findings and propose future lines of research.

## 2 Background

We consider the standard classification setting where a learning algorithm learns from the training data tuples ${\left\{x_{i},y_{i}\right\}}_{i=1}^{N}$, where *x_i_* ∈ *X* are features that can be either continuous, ordinal or categorical and $y_{i}\in C=\left\{1,\ldots ,\;m\right\}$ are discrete class labels. The goal of learning is to estimate a predictor $\hat{f}:X\to C$ that approximates the true underlying function *f: X* → *C*. The model learned, $\hat{f}$, is then used to make predictions on unseen test data ${\left\{x_{j}\right\}}_{j=1}^{M}$. For binary data, we have *y_i_* ∈  {0, 1} and without loss of generality we denote the minority class (i.e., the class with lower frequency in the training data) as the class 1. We refer to the class-specific thresholds as $\lambda_{i},\;i=1,\ldots ,\;m$. Their application to the classifier output is described below (Algorithm 1, step 2.4). We make two assumptions: (1) the probability distribution of the test data is similar to that of the training data, and (2) the class distribution of the training data provides an accurate estimate of their respective underlying prior probabilities.

### 2.1 Performance measures for imbalanced data

The commonly used measure of accuracy (correct classification rate) is a good metric when data sets are balanced. However, it can be misleading for imbalanced data. For example, the naïve strategy of classifying all the examples into the majority class would obtain 99% accuracy in a data set composed of 99% examples of this class. Therefore, other measures are necesary when dealing with imbalanced data.

Several performance measures have been proposed in imbalanced learning, all of which are computable from the elements of the confusion matrix (Table 1). Some of the most extensively used measures are:

$$\begin{array}{ccc} \text{TNR} & = & \frac{\text{TN}}{\text{TN}+\text{FP}};\;\text{Recall}\;(=\;\text{TPR})\;=\;\frac{\text{TP}}{\text{TP}+\text{FN}};\\ \;\text{Precision} & = & \frac{\text{TP}}{\text{TP}+\text{FP}};\;\text{FPR}\;=\;\frac{\text{FP}}{\text{TN}+\text{FP}} \end{array}$$

$$\text{Macro}-\text{accuracy}\;=\;\frac{\text{TPR}\;+\text{TNR}}{2};\;G-\text{mean}\;=\;\sqrt{\text{TPR}\;\times \;\text{TNR}};$$

$$F1-\text{score}\;=\;\frac{2\;\times \;\text{Precision}\;\times \;\text{Recall}}{\text{Precision}+\text{Recall}}=\;\frac{2\text{TP}}{2\text{TP}+\text{FP}+\text{FN}}$$

**Table 1:** Confusion matrix in binary classification.

	Predicted positive	Predicted negative
Actual positive	TP (true positive)	FN (false negative)
Actual negative	FP (false positive)	TN (true negative)

The macro F1-score is a widely used measure and is calculated by considering each class separately as the positive class and then averaging their corresponding F1-scores. In addition, the receiver operating characteristic (ROC) curve is often employed [6]. The ROC curve is generated by plotting the TPR (*y*-axis) and the FPR (*x*-axis) while moving through the whole spectrum of decision thresholds. The area under the ROC curve (AUROC) – generally computed numerically – is often a measure of interest that provides a summary of the ROC curve as a single number. However, ROC curves suffer from a serious limitation for evaluating performance under class imbalance. When data are highly imbalanced, ROC curves fail to capture large changes in the number of false positives (FP) since the denominator of FPR is largely dominated by TN. For this reason, precision-recall (PR) curves are preferred over ROC curves for imbalanced data [7] and are therefore our choice here. PR curves are computed by moving the decision threshold and plotting recall (*x*-axis) and precision (*y*-axis). Analogous to the ROC curve, the area under the PR curve (AUCPR) is typically employed as a summary measure.

### 2.2 Learning from imbalanced data

Many solutions have been proposed to deal with imbalanced data. These solutions mainly fall into one of the following three major strategies: (1) cost-sensitive learning, (2) rebalancing mechanisms, and (3) threshold-moving. These strategies are briefly discussed below (for a detailed account see [6], [7], [19]).

(1) *Cost-sensitive learning* places different misclassification costs on the different classes. Higher misclassification costs for the minority class can be imposed by a loss function. In fact, by altering the training class distribution, rebalancing mechanisms effectively impose different misclassification costs and can be deemed as equivalent to cost-sensitive learning [26]. For this reason, we only consider rebalancing methods in this article.
(2) *Rebalancing* mechanisms resample the data to make the training data more balanced. Such data preprocessing solutions do not require modifying the learning algorithm and therefore can be employed with existing learning algorithms.
(3) In *threshold-moving,* a model is learned from the data set with either the original or modified class proportions and its continuous output is converted into a class label by applying an appropriate threshold.

The techniques relevant to this work are discussed in the following sections.

### 2.3 Bagging ensemble

Ensemble methods use a set of classifiers to improve upon individual classifiers’ performance [27]. Two popular ensemble techniques are boosting and bagging [28]. In this work, we focus on bagged ensembles since, in general, boosted ensembles do not perform better with imbalanced data [6].

In bagging (Algorithm 1), a set of base classifiers are learned from different samples of the given training set. At the prediction time, the outputs of the base classifiers are aggregated. The main principle behind bagging's performance, given that each base classifier performs above chance, is that the averaging reduces the variance of individual classifiers without increasing their bias. Bagging generally performs well with unstable base learners for whom small changes in the training data lead to large changes in the learned model [28]. For example, decision trees (DT) and neural networks (NN) are unstable classifiers and thus suitable for bagging.

Clearly, different sampling mechanisms (Algorithm 1, step 1.2) and different thresholds (step 2.4) can be used which will yield different models and different outputs. All the methods tested here use a variation of Algorithm 1, and we will discuss their sampling mechanisms and thresholds in the next section.

Furthermore, different aggregation methods can be used to combine the outputs of the base classifiers, e.g., hard-voting to make crisp class assignments or soft-voting for probabilistic predictions. It is known that soft-voting generally provides better performance than hard-voting [21], [29], [30]. We, therefore, use soft-voting in this work (Algorithm 1, step 2.3).

### 2.4 Resampling mechanisms

In this section, we briefly describe the different resampling mechanisms that can be used in step 1.2 in Algorithm 1. One of the simplest ways to resample is to sample each instance with equal probability with replacement, i.e., a non-parametric bootstrap, as in the original bagging algorithm [28]. This sampling mechanism is not currently popular with imbalanced data as it preserves the imbalanced class distribution, which is thought to be detrimental to learning. However, we argue here that this mechanism works well when appropriate thresholds are available. Our proposed method, PT-bagging (discussed below), uses this sampling mechanism.

Commonly used resampling mechanisms for imbalanced data (here called rebalancing methods) try to balance the class proportions. Perhaps the simplest and most popular undersampling mechanism used for ensemble learning is referred to as exactly balancing (EB). EB resampling preserves the minority class instances while randomly undersampling majority class instances such that the class proportions are exactly balanced. Roughly balancing (RB) is a powerful variation of EB that improves performance by increasing diversity of the classifiers [12]. RB, like EB, preserves the minority class instances but undersamples the majority class instances as determined by a negative binomial distribution, in effect roughly balancing the class proportions. Oversampling mechanisms, on the other hand, over-sample the minority class examples. Previous studies indicate that undersampling generally performs better than oversampling [6], [31], [32]. Furthermore, undersampling is computationally more efficient than oversampling since it discards a large part of the training data.

More sophisticated hybrid methods that combine oversampling and undersampling have been proposed. One of the most popular such methods is the synthetic minority oversampling technique (SMOTE) [9], which generates new minority class examples by interpolation while undersampling the majority class examples. SMOTE often performs well in combination with a bagging ensemble. More recent methods such as Random Balance (RNB) have combined insights from both, SMOTE and RB [33]. Specifically, RNB randomly selects a class proportion and oversamples one of the classes accordingly with interpolated examples using SMOTE while the other class is undersampled. RNB aims at increasing the diversity in the base classifiers, which in turn often improves the ensemble performance. It is important to notice that the threshold of 0.5 is normally used with these rebalancing methods.

Rebalancing methods, however, present a number of potential shortcomings. An important side effect of rebalancing is that it can lead to miscalibrated posterior probability estimates, as recent studies have found [14], [15]. Another – usually unnoticed – potential problem of resampling techniques is that of the prior shift, i.e., differing training (balanced) and test (natural class proportion) distributions [15]. This might create additional problems since a model learned on a balanced data is then evaluated in a different setting. Lastly, the original density of examples is asymmetrically modified for the classes (e.g., by undersampling the majority class or oversampling the minority class), which might lead to undesired biases in the model. By contrast, the simple bootstrap sampling does not modify the data distribution, which led us to hypothesize that it will be less prone to these problems.

## 3 Probability threshold bagging (PT-bagging)

Several studies have considered threshold-moving as a method to deal with class imbalance [20], [34]. For example, Maloof [20] compared threshold-moving to RUS using a single classifier and concluded that they achieve similar performance in terms of ROC. However, to the best of our knowledge, previous studies have not considered threshold-moving in combination with bagging.

The basic idea behind PT-bagging is to leverage bagging (Algorithm 1) to first obtain well calibrated posterior estimates and appropriately threshold them afterward according to the performance measure to be maximized. PT-bagging learns base classifiers from simple bootstrap replicates of the original data set, which preserves the class distribution. Then, following Algorithm 1, probabilistic predictions for each class *k* are averaged across the base classifiers to obtain a final posterior probability estimate $\hat{\;P}\left(y=k|x\right)$ (step 2.3). To get a class label, first, the probability estimate is transformed into a score by dividing it by its respective class threshold *λ_k_* (step 2.4). The class *k* for which this score $\hat{\;P}\left(y=k|x\right)/\lambda_{k}$ is the largest is then assigned. According to the categorization proposed by Hernandez-Orallo et al. [35], PT-bagging employs a score-driven threshold, as opposed to, e.g., a fixed threshold of 0.5 used by rebalancing methods. Crucially, in PT-bagging the thresholds can be adapted to maximize a measure of interest. To maximize macro-accuracy, the optimal threshold for class *k* is equal to the class prior in the training data, i.e., $\lambda_{k}=P\left(y=k\right)$ (see Theorem 1). For instance, if the averaged posterior probability for class 0 is $\hat{\;P}\left(y=0|x\right)=0.7$, and the prior of this class is 0.8 (i.e., P(y=0)=0.8), then its score is 0.7/0.8= 0.875, and consequently the score for class 1 is 0.3/0.2 = 1.5. Thus, class 1 will be assigned even though it has a lower posterior. The calculation of this score is identical in the multiclass setting. We henceforth specify the method with the threshold that maximizes a particular measure using a subscript: PT_MA_-bagging for macro-accuracy and PT_F1_-bagging for macro F1-score. We employ the notation PT-bagging without a subscript when we refer to measures that are independent of the threshold (e.g., AUCPR and posterior probability calibration).

### 3.1 Threshold for maximizing macro-accuracy

The following theoretical result aims to provide insight into the mechanism behind the performance of threshold-moving for the macro-accuracy measure. The reader should note that the main message of Theorem 1 is not finding the optimal thresholds (or misclassification costs) for the macro-accuracy measure, which are known to be equal to the inverse of the priors, but rather a constructive proof of an algorithm that maximizes macro-accuracy for binary and multiclass data. Theorem 1’s proof shows that a necessary condition for a method to maximize macro-accuracy is to have good estimates of the posterior probabilities $\hat{\;P}\left(y=k|x\right)$ for each class *k*. To simplify notation and improve readability, we consider the binary class problem. The same proof trivially generalizes to a multiclass setting.

Theorem 1 *Proposition: Let* P(y=j) *be the prior of class j and* P(y=j|x) *be the true (unknown) posterior probability of class* *j given x. If proportions of each class are unchanged from training to test, then predicting the class k such that*

(1) $$k=\;\underset{j}{\text{argmax}\;}\frac{P\left(y=j|x\right)}{\;P\left(y=j\right)}$$

maximizes the macro-accuracy.

Proof Let C={1,0} be the class labels (positive = 1 and negative = 0). Let *d* be a random variable corresponding to the class predicted by a classifier. Thus, P(d=k|x) is the predicted probability of a model for class *k* given *x*.

We shall first derive the population expression of macro-accuracy. Recall that macro-accuracy is defined as (TPR+TNR)/2 where TPR=TP/P andTNR=TN/N. Recall that P=TP+FN and N=TN+FP. Let us first derive a continuous expression for TPR. Notice first that $\text{TP}/\left(P+N\right)=\int_{R}P\left(y=1|x\right)P\left(d=1|x\right)p\left(x\right)dx$ and that P/(P+N)=P(y=1). Thus, the ratio of the first expression over the second is equal to TPR=TP/P. That is,

$$\text{TPR}=\;\frac{\int_{R}P\left(y=1|x\right)P\left(d=1|x\right)p\left(x\right)dx}{P\left(y=1\right)}$$

The derivation of TNR is analogous. Thus, dividing the sum of TPR and TNR by 2 yields the expression of macro-accuracy:

$$\begin{array}{ccc} & & \frac{1}{2}\;\frac{\int_{R}P\left(y=1|x\right)P\left(d=1|x\right)p\left(x\right)dx}{P\left(y=1\right)}\\ & & \;+\frac{1}{2}\;\frac{\int_{R}P\left(y=0|x\right)P\left(d=0|x\right)p\left(x\right)dx}{P\left(y=0\right)} \end{array}$$

By entering both terms into the same integral:

(2) $$\frac{1}{2}\int_{R}\left\{\frac{P\left(y=1|x\right)}{\;P\left(y=1\right)}P\left(d=1|x\right)\;+\;\frac{P\left(y=0|x\right)}{P\left(y=0\right)}P\left(d=0|x\right)\right\}p\left(x\right)dx$$

Therefore, maximizing the integral in (2) is equivalent to asking for the optimal choice of P(d=1|x) and P(d=0|x) for a given *x* – in other words, how to assign class labels 1 or 0 in a wise way (perhaps probabilistically), given *x*. Notice that the bracket inside the integral in (2) is nothing but a convex combination:

(3) $$\frac{P\left(y=1|x\right)}{P\left(y=1\right)}\beta_{x}+\;\frac{P\left(y=0|x\right)}{\;P\left(y=0\right)}\left(1-\beta_{x}\right)$$

where we defined $\beta_{x}:=P\left(d=1|x\right)$. Thus, by monotonicity, the convex combination (3) is maximized at *x* if and only if we place probability 1 to the largest term. That is to say, an optimal method assigns the positive class 1 with probability 1 if the term P(y=1|x)/P(y=1) is the largest or assigns the negative class with probability 1 if P(y=0|x)/P(y=0) is the largest. That is,

(4) $$\beta_{x}:=P\left(d=1|x\right)=\left\{ \begin{array}{c} 1,\;if\;\frac{P\left(y=1|x\right)}{P\left(y=1\right)}>\frac{P\left(y=0|x\right)}{P\left(y=0\right)}\;\\ 0,\;Otherwise \end{array}\right.$$

This is indeed the method proposed above, in Eq. (1). ****□

Critically, we note that the optimal method of Eq. (4) will not have the true P(y=1|x) at hand but an estimation $\hat{\;P}\left(y=1|x\right)$ instead. Notice also that all the other quantities needed to make a decision in (4) are known constants, i.e., P(y=1) and P(y=0) (i.e., the thresholds). Therefore, the good performance of the method totally relies on having good posterior probability estimates$\hat{\;P}\left(y=1|x\right)$.

### 3.2 Threshold for maximizing macro F1-score

Unlike macro-accuracy, there is no closed-form expression for a threshold that maximizes the macro F1-score [25]. In general, increasing the threshold increases the precision of the minority class at the expense of decreased recall. It is, however, known that 0.5 is the upper bound on the optimal threshold for the F1-score [25]. In the absence of any additional information and tuning, we set the threshold for the minority class to (P(y=1)+0.5)/2. The rationale behind this threshold is that it is set midway between the threshold for maximizing the average recall (i.e., the training set class prior) and the upper bound on the threshold for maximizing the F1-score (i.e., 0.5).

Note that methods have been proposed to estimate the optimal threshold for maximizing the F1-score but they require additional data for fine-tuning and are susceptible to the Winner's curse [25]. Using tuning methods is difficult for most of the datasets used here, as they are relatively small. Importantly, our aim here was to test a tuning-free threshold. However, finding better thresholds, when possible, can conceivably result in further improvements.

The reader should note that the proposed threshold is defined for the binary class setting. Its extension to the multiclass setting will be considered in future work.

## 4 Experimental setup

We used the R statistical environment (http://www.r-project.org/) with corresponding packages and default parameters unless otherwise specified.

Selecting a proper base classifier learning algorithm (Algorithm 1, step 1.3) is crucial as our method relies on good posterior probability estimates at the test time. Previous work has shown that bagged probabilistic decision trees (DT) provide good posterior probability estimates [22]. These are, in fact, more reliable than other classifiers such as logistic regression [22]. Here, we employ unpruned J48 decision trees, an implementation of the C4.5 trees available in the “RWeka” package [30], [36]. Even though it is common to apply Laplace smoothing to the leaf probabilities of the individual decision trees, it can be detrimental for imbalanced data [37]. Therefore, we did not use Laplace smoothing.

In order to evaluate the generality of our method, we also employed neural networks (NN) as base classifiers. Bagged neural networks are known to offer well calibrated posterior probability estimates [23], and thus we hypothesized that this would be a suitable choice for our method. We used a single hidden layer with logistic units and softmax output, implemented with the “nnet” package. Following a rule of thumb, we set the number of hidden units to 2/3 of the input dimension plus the number of classes [38].

We studied the effect of the number of base classifiers by varying them in {5, 10, 15, 25, 50, 100}. We ran 5 × 2-fold cross-validation for each method on each data set. The Friedman test was used to test if there were differences across the methods and if the test passed at 95% significance, a posthoc Nemenyi test was performed to identify any pairwise differences [39]. A paired Wilcoxon rank sums test was used to compare two methods directly.

### 4.1 Datasets

We used 36 imbalanced binary data sets (Table 2): 14 from the HDDT repository (http://www3.nd.edu/∼dial/hddt/), 19 from the KEEL repository (http://sci2s.ugr.es/keel/imbalanced.php), and three from the UCI repository (https://archive.ics.uci.edu/ml/datasets.html). Note that the evaluation with neural network ensembles includes 26 data sets that contain only numerical attributes. Only the complete instances of data were used and any constant attributes were removed. Table 2 shows a summary of the datasets used in our experiments. The attributes of the data sets are numerical, categorical or numerical-categorical mixed. For the multiclass setting, we used 15 data sets from the KEEL repository (Table 3).

**Table 2:** Overview of the binary data sets obtained from UCI, HDDT* and KEEL† repositories (names were shortened for convenience).

Dataset	#Inst	#Attr	#Num	%Min	Dataset	#Inst	#Attr	#Num	%Min
pima	768	8	8	34.5	br-y†	277	9	0	29.2
ion	351	34	34	35.9	cl0vs4†	173	13	13	7.5
sonar	208	60	60	46.6	ecoli4†	336	7	7	5.9
spectf*	267	44	44	20.6	hab†	306	3	3	26.5
phon*	5404	5	5	29.3	led7_xvs1†	443	7	7	8.3
page*	5473	10	10	10.2	pb-1-3vs4†	472	10	10	5.9
ism*	11,180	6	6	2.3	shut-0vs4†	1829	9	9	6.7
letter*	20,000	16	16	3.9	vow0†	988	13	13	9.1
satim*	6430	36	36	9.7	yst-2vs4†	514	8	8	9.9
compu*	13,657	20	20	3.8	yst4†	1484	8	8	3.4
segm*	2310	19	19	14.3	glass6†	214	9	9	13.5
oil*	937	49	49	4.4	new-th1†	215	5	5	16.3
estate*	5322	12	12	12	wisc†	683	9	9	34.5
hypo*	2000	24	6	6.1	car-gd†	1728	6	0	4
boun*	3505	175	0	3.5	flare-F†	1066	11	0	4
cred*	1000	20	7	30	kdd†	1642	41	26	3.2
hrt-v*	133	9	4	23.3	veh0†	846	18	18	23.5
ab9-18†	731	8	7	5.7	w-red-4†	1599	11	11	3.3

**Table 3:** Overview of the multiclass data sets, all from the KEEL repository.

Dataset	#Inst	#Attr	#Num	%Min	#Class
ontraceptive	1473	9	6	22.6	3
dermatology	366	34	34	5.5	6
balance	625	4	4	7.8	3
penbased	1100	16	16	9.5	10
shuttle	2175	9	9	0.09	5
wine	178	13	13	27	3
yeast	1484	8	8	0.3	10
pageblocks	548	10	10	0.6	5
thyroid	720	21	21	2.4	3
ecoli	336	7	7	0.6	8
autos	159	25	15	1.9	6
glass	214	9	9	4.2	6
new-thyroid	215	5	5	13.9	3
hayes-roth	132	4	4	22.7	3
lymphography	148	18	3	1.3	4

### 4.2 Methods used for comparison

For the binary class setting, we included EB-bagging as the baseline method along with RB-bagging, SMOTE-bagging and RNB-bagging as state-of-the-art methods (see Section 2.4 for details). The parameters of SMOTE were set to the commonly used setting of 500% oversampling of the minority class with 5 nearest neighbors and 100% sampling of the majority class. To investigate the benefit of using an ensemble in PT-bagging, we additionally included a single classifier (denoted as “single”) that used the complete training data to learn, and employed the same threshold-moving mechanism as PT-bagging and identical parameters as its base classifiers.

For completeness, we compared Platt scaling [40] and PT-bagging on binary data sets. Platt scaling is a well-known posthoc calibration method that transforms continuous model outputs into (calibrated) probability estimates. It firstly fits a logistic regression model to the outputs with the class labels as the dependent variable. To avoid over-fitting, we used data from 3-fold cross-validation with transformed class labels derived from the training set, as in the original paper [40]. This logistic model was then applied to the test set outputs to obtain calibrated probabilities. A threshold of 0.5 was finally applied to the calibrated probabilities to obtain the test class labels. If the posteriors are indeed calibrated after Platt scaling then this setting should yield good performance. We deemed its inclusion as relevant since, like our method, Platt scaling is an easy-to-apply *a posteriori* correction of the model outputs.

### 4.3 Performance evaluation

We evaluate the methods on three performance measures: area under the PR curve (AUCPR), macro-accuracy and macro F1-score (see Section 2.1 for details). Note that AUCPR is computed using the posterior probability estimates while the other two measures require class label assignments.

The methods considered here differ in two important aspects: (1) the resampling mechanism and (2) the threshold used. Each resampling mechanism may require its own threshold to achieve optimal performance. However, often a standard threshold is used in practice (e.g., 0.5 for EB- and RB-bagging). To evaluate the performance independently of the threshold, we devised a novel scheme called the full potential. The full potential of a method is defined as the best performance achieved over all possible thresholds on the test set. A method's performance close to its full potential means that the threshold used is more attuned to the optimal threshold for that method. We approximated the full potential by searching the optimal threshold over a grid from 0 to 1 in steps of 0.01.

Finally, we used the stratified Brier score (mean squared error) to evaluate the calibration of the posterior probabilities. The Brier score of class *k* is calculated as the average squared differences between the estimated probability of the examples from class *k* (i.e. $\hat{\;P}\left(y=k|x_{i}\right)$) and a perfectly confident probabilistic prediction (i.e., 1):

(5) $$BS^{k}=\;\frac{1}{N_{k}}\sum_{y_{i}^{*}=k}{\left(1-\hat{\;P}\left(y=k|x_{i}\right)\right)}^{2}\;$$

where *N_k_* is the number of examples of class *k* and $y_{i}^{*}=k$ refers to those examples for which *k* is the true class label. Averaging the Brier score of all the classes gives the stratified Brier score. The stratified Brier score is more appropriate when there is class imbalance since it gives equal importance to all the classes and thus allows any miscalibration of the minority classes to be spotted [41].

## 5 Results and discussion

In this section, we compare the performance of the proposed method with other state-of-the-art methods and provide further empirical insights. For brevity, when ensembles of neural networks performed similarly to those of decision trees, the results from neural networks ensembles are omitted. Unless otherwise indicated, we report results on ensembles with 100 base classifiers.

### 5.1 Binary data sets

**Fig. 1:**
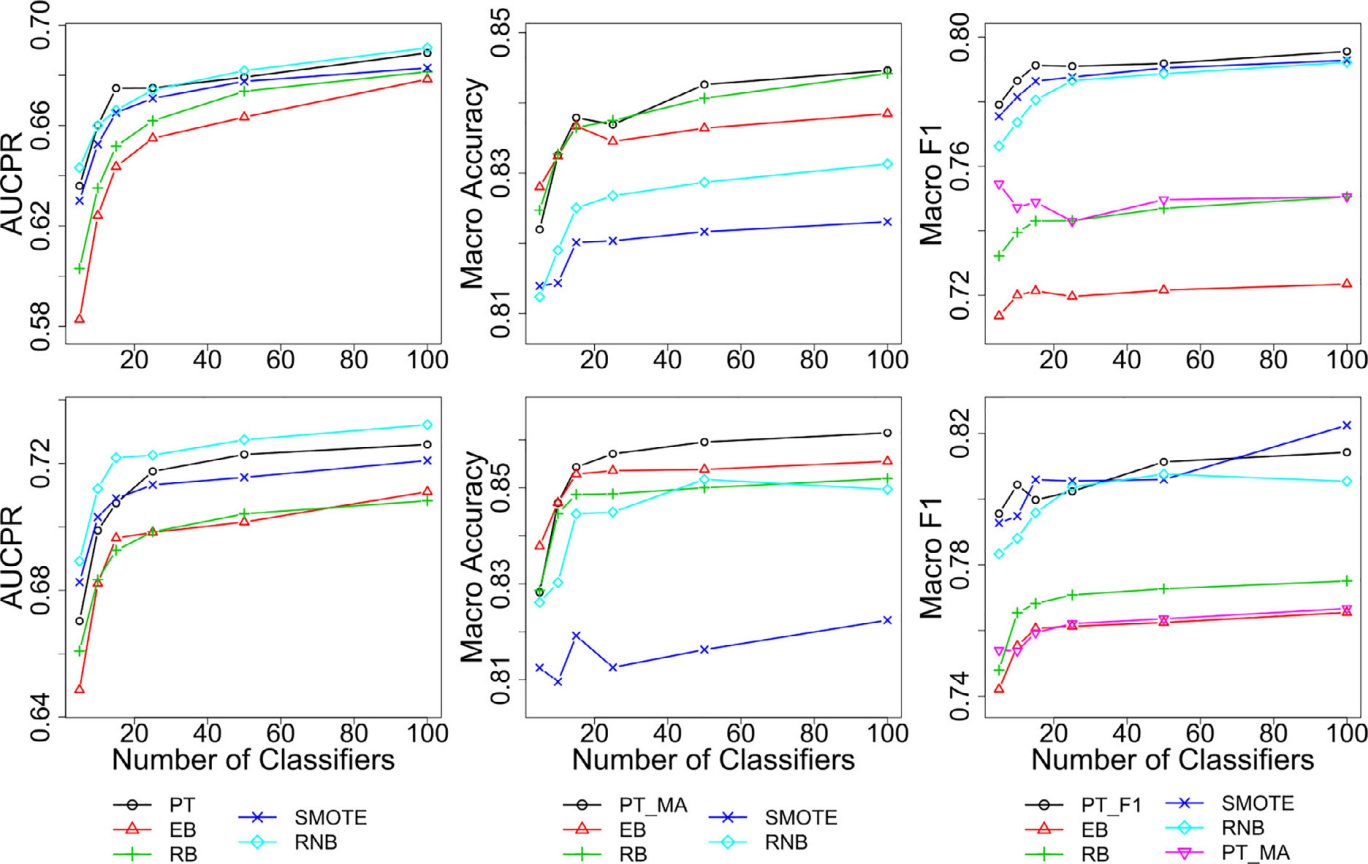
Average test performance across datasets for different numbers of classifiers in AUCPR (left), macro-accuracy (middle) and macro F1 (right). The first row shows results for DT ensembles and second row for NN ensembles. The interpolated lines are shown for convenience.

We first investigated the effects of the ensemble size (Fig. 1). It is worth mentioning that, unsurprisingly, the area under the ROC curve showed a much more cluttered picture, which we omit in the interest of space.

Overall, the following general observations can be made: (1) all methods show improvement with increasing ensemble size in all performance measures. (2) PT_MA_-, RB- and EB-bagging perform better on macro-accuracy while PT_F1_-, SMOTE- and RNB-bagging perform better on the macro F1-score. This shows that different resampling mechanisms are suitable for different performance measures; (3) PT-bagging – with appropriate thresholds – performed well in each of the evaluated measures, while the rest of methods performed poorly in at least one of them, e.g., RB-bagging performed poorly in macro F1-score and AUCPR, while SMOTE- and RNB-bagging performed poorly in macro-accuracy. Finally, (4) as expected, EB- and RB-bagging performed similarly (although RB-bagging generally fared better with decision trees) and RNB- and SMOTE-bagging did not differ significantly. We discuss these results in more detail below.

#### 5.1.1 The area under the precision-recall curve (AUCPR)

As can be readily observed, PT-, SMOTE- and RNB-bagging showed, on average, overall better performance than EB- and RB-bagging on AUCPR for all ensemble sizes (Fig. 1, left). This indicates that PT-, SMOTE- and RNB-bagging are generally able to achieve comparatively higher average precision and recall (F-measures). The Friedman test revealed a significant difference between the methods (*P* < 3e − 12, for both DT and NN ensembles).

The posthoc pairwise Nemenyi tests with DT ensembles showed a nearly significant difference between PT-bagging and EB-bagging (*P* ≈ 0.062), and between RNB- and EB-bagging (*P* ≈ 0.083). Note that lower AUCPR generally implies a lower potential for the F-measures, irrespective of the threshold used. Thus, when using DT ensembles, methods based on undersampling alone (i.e., EB- and RB-bagging) are likely to have a lower F1-score (as shown below). The single classifier clearly showed a lower AUCPR than the PT-bagging and rest of the methods (*P* < 1.5e − 6).

With NN ensembles, RB-bagging was significantly (or nearly significantly) worse than PT-, SMOTE- and RNB-bagging (*P* ≈ 0.058, 0.065 and 0.021, respectively). All the methods performed significantly better than the single classifier (*P* < 0.001). No other significant differences were found.

The choice of base classifiers (either DT or NN) did not make a significant difference in any of the method's performance (*P* > 0.17 for all Wilcoxon tests on 26 data sets). This aligns with our claim that PT-bagging can be used with different choices of base classifiers.

#### 5.1.2 Macro-accuracy

Fig. 1 (middle) shows that resampling methods offer similar or higher macro-accuracy compared to PT_MA_-bagging when a small number of classifiers were employed. This aligns with Maloof's [20] findings which showed that undersampling and threshold-moving perform similarly in terms of ROC and macro-accuracy when a single classifier is employed. However, the performance of PT_MA_-bagging improved substantially with larger ensembles (25 base classifiers or more), indicating that once the variance is reduced through bagging, the error that is left comes mostly from the bias (since bagging reduces variance but not bias). This result suggests that the bias of PT_MA_-bagging is lower than that of the other methods.

PT_MA_-bagging showed the highest number of wins as well as fewer losses compared to other methods (Table 4, left), especially with DT, where it obtained almost twice as many wins as losses against the second best performing method (RB-bagging). The Friedman test revealed a significant difference between the methods (*P* < 1e − 11 for both, DT and NN ensembles).

**Table 4:** Win/Tie/Loss tables. Each element expresses how many times the method in the row wins/ties/loses against the method in the column. The top tables show results with DT methods, and the bottom tables show results with NN methods.

	Macro-accuracy						Macro F1-score				
	EB	RB	SMOTE	RNB	Single		EB	RB	SMOTE	RNB	Single
PT_MA_	27/0/9	23/1/12	24/1/11	23/1/12	31/2/3	PT_F1_	33/0/3	31/1/4	19/2/15	19/1/16	30/0/6
EB	–	6/4/26	20/2/14	19/1/16	31/0/5	EB	–	2/2/32	1/1/34	1/2/33	10/0/26
RB	–	–	24/1/11	23/4/9	33/0/3	RB	–	–	5/2/29	6/2/28	15/0/21
SMOTE	–	–	–	15/4/17	30/1/5	SMOTE	–	–	–	18/2/16	28/0/8
RNB	–	–	–	–	34/1/1	RNB	–	–	–	–	30/0/6
	EB	RB	SMOTE	RNB	single		EB	RB	SMOTE	RNB	single

PT_MA_	13/1/12	16/0/10	20/0/6	15/2/9	26/0/0	PT_F1_	20/2/4	20/0/6	14/1/11	11/4/11	24/0/2
EB	–	15/2/9	19/1/6	14/1/11	26/0/0	EB	–	4/0/22	4/1/21	3/1/22	12/0/14
RB	–	–	19/1/6	13/1/12	26/0/0	RB	–	–	6/2/18	5/2/19	13/0/13
SMOTE	–	–	–	4/2/20	22/0/4	SMOTE	–	–	–	13/2/11	23/1/2
RNB	–	–	–	–	26/0/0	RNB	–	–	–	–	22/0/4

For DT ensembles, the posthoc pairwise Nemenyi tests showed that PT_MA_-bagging performed significantly better than EB-, SMOTE- and RNB-bagging (all *P* < 0.03) and the single classifier (*P* < 1e − 10). None of the remaining differences were significant.

NN ensembles showed a similar trend of pairwise differences as the DT ensembles, although in this case PT_MA_-bagging was only significantly better than SMOTE-bagging (*P* ≈ 0.021) and the single classifier (*P* < 1.7e − 9). It should be noted, however, that it was more difficult to obtain statistical significance with NN ensembles as fewer data sets were used.

It is also worth noting that the type of the base classifier had no significant effect on a method's performance (Wilcoxon test, *P* > 0.8 for all comparisons of a method using DT against the same method using NN) except for the single classifier which performed better with DT than with NN (*P* ≈ 0.01).

**Fig. 2:**
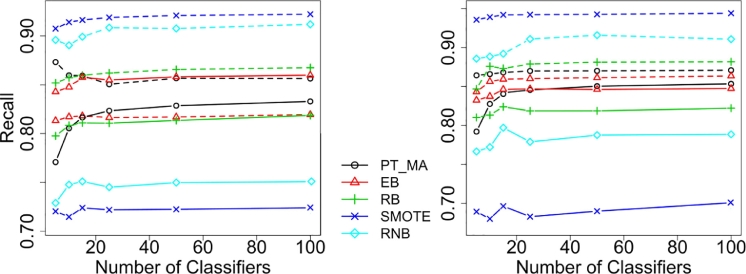
Average recall across data sets for different numbers of classifiers, separated for the minority class (solid line) and the majority class (dashed line). The left plot shows results for DT ensembles and right plot for NN ensembles.

*Symmetry of class recalls:* In order to gain insight into the bias of the methods toward predicting either class we tested the null hypothesis that, for each method, the difference between the class recalls (Fig. 2), i.e., the two components of the macro-accuracy, is equal to zero using a one sample *t*-test. With DT ensembles, the null hypothesis was rejected for all methods (*P* < 0.003) except for PT_MA_-bagging (*P* ≈ 0.18). With NN ensembles, a similar symmetry was observed for PT_MA_-bagging (*P* ≈ 0.42). In this case, EB-bagging was the only other method that did not show significantly asymmetric class recalls (*P* ≈ 0.46).

These results suggest that PT_MA_-bagging is not biased toward either class. Interestingly, only EB-bagging (using DT) showed higher recall for the minority class (Fig. 2, left), suggesting a possible overcompensation for the minority class due to the undersampling.

*Full potential*: We then tested the full potential of the methods for macro-accuracy, i.e., which we define as their maximum macro-accuracy that would be attainable if the optimal threshold for each test set were known. The Friedman test revealed a significant difference between methods (*P* < 0.001 both DT and NN ensembles).

Post-hoc pairwise comparisons with DT ensembles revealed that SMOTE-bagging (*P* ≈ 0.04) and PT_MA_-bagging (*P* ≈ 0.1) have (respectively) a significantly and nearly-significantly higher full potential than EB-bagging. Again, PT-bagging and the rest of the ensemble methods clearly outperformed the single classifier in terms of potential (*P* < 1e − 10). The rest of the pairwise differences were not significant.

With NN ensembles, the trend changed for RB-bagging, now exhibiting generally lower potential for macro-accuracy than the other methods, with significantly (or nearly significantly) lower potential than RNB- and SMOTE-bagging (*P* ≈ 0.031 and *P* ≈ 0.081, respectively). This drop in the full potential of RB-bagging when using NN can also be appreciated from its lower averaged AUCPR in Fig. 1 as compared to, for example, EB-bagging. This seems to suggest that RB-bagging might not generalize well across base classifier choices, conceivably because it might be leveraging properties of decision trees. To our knowledge, no other studies have analyzed the performance of RB-bagging with base classifiers other than DT and thus more analyses are needed.

Finally, the single classifier showed lower potential than PT-bagging (*P* < 8e − 7) and the rest of the ensembles (*P* < 0.002).

To shed light on how well-tuned the thresholds employed for each method were, we compared their actual and full potential macro-accuracy. The average absolute difference between the full potential macro-accuracy and the actual macro-accuracy was calculated across cross-validation folds and datasets (Table 5, left). PT_MA_-bagging performed closer to its full potential than the other ensemble methods. This suggests that the prior-based threshold employed by PT_MA_-bagging is a close-to-optimal choice. The single classifier performed closest to its full potential macro-accuracy, however its full potential was markedly lower than the other methods – as discussed above – yielding thus a lower performance (Table 4).

**Table 5:** The average difference between the actual performance and full potential. The first row shows results for DT methods and second row for NN methods.

	Macro-accuracy						Macro F1-score					
	PT_MA_	EB	RB	SMOTE	RNB	single	PT_F1_	EB	RB	SMOTE	RNB	single
DT	1.9%	2.5%	2.3%	4.7%	3.4%	0.9%	2.6%	9.5%	6.9%	2.9%	3.2%	1.1%
NN	1.7%	2.0%	2.1%	5.9%	3.3%	0.6%	2.9%	6.5%	5.5%	2.9%	3.6%	0.9%

#### 5.1.3 Macro F1-score and plug-in potency

An important advantage of our method is that a learned ensemble can be used to make predictions that optimize any measure of interest by applying an appropriate threshold *a posteriori*. We applied the proposed threshold to maximize the macro F1-score (see Section 3.2) to the outputs of the same PT-bagging ensembles used above. The resulting method is termed PT_F1_-bagging (Fig. 1, right).

The Friedman test revealed a significant difference between the methods for DT (*P* < 0.0008; *P* < 0.1 for NN). Post-hoc tests showed that PT_F1_-bagging had a significantly higher macro F1-score than PT_MA_-bagging, EB-bagging, and RB-bagging (all *P* < 0.0008) for both DT and NN ensembles. However, with either choice of the base classifier, PT_F1_-bagging was not significantly different from RNB- or SMOTE-bagging. As in the previous measures, the single classifier performed markedly worse than PT_F1_-, SMOTE- and RNB-bagging (*P* < 0.002) with either base classifier, while not worse than EB-bagging with NN (*P* ≈ 0.99) and not worse than RB-bagging with both NN and DT (*P* > 0.91).

Again, none of the methods showed significant differences when comparing their performances with DT and NN ensembles (*P* > 0.17 for all Wilcoxon tests).

*Full potential*: We found significant differences between the methods in terms of the full potential of the macro F1-score (Friedman test, *P* < 0.055 for both DT and NN). With DT ensembles, pairwise posthoc tests revealed only one trend level difference between PT_F1_- and EB-bagging (*P* ≈ 0.1) in favor of PT_F1_-bagging. With NN ensembles, as noted above with macro-accuracy, RB-bagging's potential generally decreased showing a significantly lower full potential than PT_F1_-bagging (*P* ≈ 0.04). As expected, the single classifier had significantly lower full potential than all the ensembles with either type of base classifier (*P* < 0.0022). No other differences were significant.

Finally, the averaged differences between the actual and the full potential macro F1-score show that PT_F1_-bagging – which uses our novel threshold – performed closer to its full potential than the rest of ensemble methods (Table 5, right). Also, SMOTE- and RNB-bagging performed closer to optimal than EB- and RB-bagging. Additionally, the single classifier performed closest to its optimal performance, yet again, its full potential and actual performance were markedly lower than the ensemble methods (Table 4).

Taken together, these results imply that the methods that exhibit a performance clearly lower to that of their full potential could do better if proper thresholds could be found, which is often not possible without using computationally expensive tuning procedures. Overall, the results above support our claim that PT-bagging passes as a plug-in method where the threshold can be set *a posteriori* according to the performance measure of interest.

#### 5.1.4 Posterior probability calibration

So far, we have shown that PT-bagging performs competitively on three different measures. In particular, good performance in the macro F1-score and macro-accuracy (Theorem 1) is only possible if posterior probabilities are well calibrated. In the following, we argue that PT-bagging estimates well calibrated posterior probabilities.

An empirical study by Niculescu-Mizil and Caruana [23] showed that bagged DT and NN ensembles estimate well calibrated posterior probabilities, making additional calibration – e.g., with Platt scaling – unnecessary. However, probability calibration is a relatively understudied problem for imbalanced data. In this direction, a recent study proposes to correct the calibration for undersampling [14] and another study proposes the use of an undersampling-based variation of Platt scaling to obtain calibrated probabilities [42]. These studies use the (stratified) Brier score (see Eq. (5)) to quantify calibration. Wallace and Dahabreh [41] found that undersampling combined with bagging leads to a lower Brier score for the minority class, i.e., a better calibration for this class, without sacrificing the overall Brier score. We found similar results in our experiments with both DT and NN ensembles. Specifically, the rebalancing methods showed a significantly lower Brier score for the minority class than PT-bagging and the single classifier, while PT-bagging fared better with the majority class than the rebalancing methods and the single classifier (Friedman test *P* < 2e − 16; all pairwise posthoc Nemenyi tests *P* < 0.018 for both DT and NN based methods).

This seemingly negative result for PT-bagging, i.e., a higher Brier score for the minority class than the majority class, can be attributed to a potential shortcoming of the stratified Brier score. The Brier score for class *k* decreases with crisp posteriors, e.g., the Brier score for the minority class would become zero if all posterior probabilities for this class were 1 (see Eq. (5)). Thus, the information in the non-crisp posteriors is ignored by the Brier score and overestimated probabilities will, wrongly, lead to a lower Brier score. This suggests that the stratified Brier score might not be appropriate for qualifying posterior calibration over imbalanced data, as it is not necessarily indicative of other performance measures. Developing new measures to quantify calibration is beyond the scope of this paper.

**Fig. 3:**
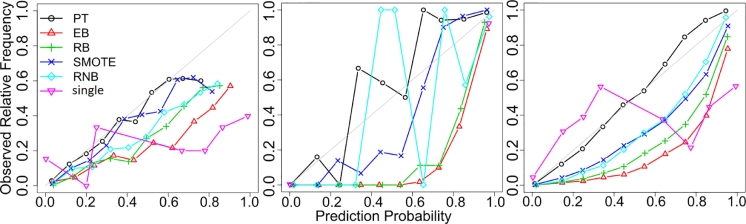
Reliability plots for DT ensembles with 100 classifiers; spectf (UCI, left), pb-1-3vs4 (KEEL, middle) and satim (HDDT, right). We used 10 bins to discretize the posterior probability for the minority class $\hat{\;P}\left(y=1|x\right)$ (*x*-axis) for all five runs and two folds. The corresponding observed frequencies of the minority class (*y*-axis) were calculated for each bin (i.e., the “true” P(y=1|x)). A method lining up with the diagonal is well calibrated while values below the diagonal are overestimating the probability of the minority class.

Reliability plots provide an alternative visual way to evaluate calibration quality [23], overcoming the aforementioned deficiencies. Three examples of reliability plots are shown in Fig. 3. Visual inspection revealed that PT-bagging probabilities were well calibrated (i.e., close to the diagonal line) for the majority of the data sets (21 out of 36 DT ensembles, and 15 out of 26 NN ensembles; see Supplementary material). In contrast, all the other methods tended to systematically overestimate the posteriors for the minority class (hence their lower Brier scores). Moreover, whenever PT-bagging estimated miscalibrated posteriors (i.e., not aligned with the diagonal), the other methods failed too. These results suggest that PT-bagging estimates relatively well-calibrated posteriors.

#### 5.1.5 Comparison with Platt scaling

To investigate the effect of direct calibration, we applied Platt scaling to the posterior probabilities of the ensembles with 100 classifiers used above, followed by a threshold of 0.5. With the DT ensembles, PT_MA_-bagging outperformed Platt scaling in macro-accuracy (Paired Wilcoxon test, *P* < 1e − 8; Win/Tie/Loss = 34/0/2). Platt scaling performed relatively better in the macro F1-score. Nevertheless, PT_F1_-, SMOTE- and RNB-bagging still outperformed Platt scaling (Paired Wilcoxon test, *P* < 0.08 in all comparisons).

Results with NN ensembles showed a similar picture for the macro-accuracy measure, where PT_MA_-bagging clearly outperformed Platt scaling (*P* < 1e − 6; Win/Tie/Loss = 21/1/4). However, in this case, the differences between Platt scaling and PT_F1_-, SMOTE- and RNB-bagging were non-significant (all *P* > 0.4).

In conclusion, probability calibration using Platt scaling did not provide an improvement over the methods investigated in this article. This corroborates previous results showing that posterior calibration for bagged DT and NN ensembles is often unnecessary as their probability estimates are generally well calibrated [23].

### 5.2 Multiclass data sets

An important advantage of our method is that it can be directly extended to the multiclass setting. For multiclass data, the threshold-moving technique can be applied by dividing the posteriors by appropriate probabilities (see Theorem 1). For instance, to maximize macro-accuracy, the thresholds of PT_MA_-bagging are set equal to the prior probabilities of the respective class (see Section 3.1 and Algorithm 1). We evaluated this approach on 15 multiclass data sets (Table 3). For comparison, we used the UnderBagging to OverBagging method (UnderOver) [43]. This method uses under- or over-sampled instances of different classes in proportion to the majority class size controlled by a parameter *a* which corresponds to the sampling rate of the largest class. The parameter *a* changes the ensemble from UnderBagging (*a* = 0) to OverBagging (*a* = 100). Notice that UnderOver (*a* = 0) is the multiclass extension of EB-bagging in which the majority classes are undersampled to match the least frequent class. We varied the parameter *a* in {0, 10, 25, 50, 100}.

**Algorithm 1:** Pseudo-code for the bagging ensemble.

1. Learning:
1.1. Input: A training set *S*$={\left\{\left(x_{i},\;y_{i}\right)\right\}}_{i=1}^{N};$$y_{i}\in C=\left\{1,\ldots ,\;m\right\}$, where *m* is the number of class labels and *n* the number of base classifiers.
1.2. Generate *n* training data sets by sampling^1^*S*.
1.3. Learn *n* base classifiers from each sample.
2. Prediction:
2.1. Input: an instance *x, n* base classifiers, a probability threshold *λ_k_* for each class *k*.
2.2. Each base classifier *i* gives a probabilistic estimate ${\hat{\;P}}_{i}\left(y=k|x\right)$ for each label k∈{1,…,m} given a test instance *x*.
2.3. Compute averages of probabilistic predictions for each class k∈{1,…,m}: $\hat{\;P}\left(y=k|x\right)=\;\frac{1}{n}\sum_{i}^{n}{\hat{\;P}}_{i}\left(y=k|x\right)$.
2.4. Rank each class k∈{1,…,m} according to:$\;\hat{\;P}\left(y=k|x\right)/\lambda_{k}$.
2.5. Assign the label for which the score in 2.4 is the highest.

^1^The sampling mechanism has been purposefully left unspecified. It is specified in the context of the respective methods.

We used similar experimental settings to those used for the binary data sets, i.e., simple bootstrap sampling for PT-bagging and 5 × 2-fold cross-validation. Following their good performance in binary data sets, here we employed only DT ensembles of size 100.

As there are many methods (PT-bagging and five competing methods) relative to the number of data sets, the Friedman test is unlikely to reveal differences. Therefore, here we only performed pairwise tests. Firstly, we selected the single competitor method with the highest average macro-accuracy. This method – with an average macro-accuracy of 0.756 – was UnderOver-bagging (with *a* = 50). This method and PT_MA_-bagging (average macro-accuracy 0.789) showed a trend-level difference (Paired Wilcoxon test, *P* ≈ 0.0946). Furthermore, of the total 15 multiclass data sets, PT_MA_-bagging had eight overall wins against the five competitors, while the next best method was UnderOver-bagging (with *a* = 10) with four wins. This result suggests that PT-bagging can be successfully employed for multiclass data sets when appropriate thresholds are available, although further tests are needed to confirm stringent statistical significance.

## 6 Conclusions and future work

We proposed a simple plug-in method, PT-bagging, for imbalanced classification. Our method relies on simple bootstrap sampling – which preserves the natural class distribution – to create a bagging ensemble followed by threshold-moving to assign class labels. Our results and analyses showed that PT-bagging, unsurprisingly, outperforms the single classifier baseline and performs competitively to four state-of-the-art ensemble methods. Furthermore, it does so in three performance measures: AUCPR, macro-accuracy and macro F1-score*.* We showed that the class priors provide the optimal thresholds for maximizing the macro-accuracy measure, and we introduced a new intuitive threshold for maximizing the macro F1-score and demonstrated its effectiveness.

We showed that PT-bagging (combined with an appropriate threshold when needed) performs at least as well as the best competitor method in each of the three performance measures and does not underperform in any of them. Critically, it does so by reusing the same ensemble models across performance measures. By contrast, all other methods proved to be weak in at least one of the three measures. We observed that the undersampling-only methods (EB- and RB-bagging) were more suitable for maximizing the macro-accuracy, while methods combining synthetic oversampling with undersampling (SMOTE- and RNB-bagging) fared better with the macro F1-score. Thus avoiding the necessity of choosing an arbitrary threshold or identifying a measure-specific threshold.

Our analysis provided several additional insights. Specifically, we found: (i) PT-bagging is less biased toward either class than other methods, (ii) it performs close to its full potential, (iii) it performs well with different choices of base classifier, (iv) PT-bagging can be directly extended to multiclass data when appropriate thresholds are available; and finally, (v) a potential shortcoming of the Brier score in quantifying probability calibration and.

Taken together, our work provides a competitive and simple alternative to other rebalancing- and synthetic oversampling-based ensemble methods, which are often the first choice to deal with class imbalance. We hope that our results and analyses will increase interest in the threshold-moving technique and provide a basis for developing new threshold-based methods for imbalanced classification.

An important but understudied question is whether to use the *natural* class distribution for learning [19]. Weiss and Provost [16] studied this question empirically and concluded that generally a different class distribution leads to better performance. Our results stand in contrast to their conclusion. An important difference between their procedures and ours, which can at least partially explain, the different conclusions, is the use of a single decision tree versus an ensemble. As our results show, a single classifier as well as simple bootstrap bagged small ensembles performs poorly, but the performance improves with the ensemble size. Considering this, we conclude that a large enough bagging ensemble successfully models the data with its natural class distribution.

We can take several possible directions in the future. One such possible direction would be to use intrinsic data properties to improve the sampling mechanism (see, e.g., [7]). Such methods can leverage properties of the data and help with difficult situations, e.g., small disjuncts of the minority class. Additionally, as our method avoids computationally expensive retraining, we aim to investigate the suitability of our method in environments with dynamic costs.

## Appendix Supplementary materials

Image, application 1

Image, application 2
